# Hypoxia and Multilineage Communication in 3D Organoids for Human Disease Modeling

**DOI:** 10.3390/biomimetics10090624

**Published:** 2025-09-16

**Authors:** Seif Ehab, Ola A. Gaser, Ahmed Abdal Dayem

**Affiliations:** 1 Zoology Graduate Program, Department of Zoology, Faculty of Science, Cairo University, Giza 12613, Egypt; 2 Wake Forest Institute for Regenerative Medicine, Wake Forest School of Medicine, Winston-Salem, NC 27109, USA; 3 Stem Cell and Regenerative Biotechnology Major, School of Advanced Biotechnology, College of Institute of Science and Technology, Molecular & Cellular Reprogramming Center, Institute of Advanced Regenerative Science, Institute of Health, Aging & Society, Konkuk University, Seoul 05029, Republic of Korea

**Keywords:** hypoxia, multilineage, oxygen tension, organoids, hypoxia-inducible factors

## Abstract

Organoids, self-organizing, three-dimensional (3D) multicellular structures derived from tissues or stem cells, offer physiologically relevant models for studying human development and disease. Compared to conventional two-dimensional (2D) cell cultures and animal models, organoids more accurately recapitulate the architecture and function of human organs. Among the critical microenvironmental cues influencing organoid behavior, hypoxia and multilineage communication are particularly important for guiding cell fate, tissue organization, and pathological modeling. Hypoxia, primarily regulated by hypoxia-inducible factors (HIFs), modulates cellular proliferation, differentiation, metabolism, and gene expression, making it a key component in disease modeling. Similarly, multilineage communication, facilitated by intercellular interactions and extracellular matrix (ECM) remodeling, enhances organoid complexity and immunological relevance. This review explores the dynamic interplay between hypoxia and multilineage signaling in 3D organoid-based disease models, emphasizing recent advances in engineering hypoxic niches and co-culture systems to improve preclinical research fidelity. We also discuss their translational implications for drug screening, regenerative medicine, and precision therapies, while highlighting current challenges and future opportunities. By integrating biophysical, biochemical, and computational approaches, next-generation organoid models may be further optimized for translational research and therapeutic innovation.

## 1. Introduction

Organoids, three-dimensional (3D) multicellular microtissues derived from stem cells or tissues, have revolutionized the study of human biology and disease [1]. These self-organizing structures closely mimic the architecture and function of human organs, providing a more physiologically relevant model than traditional two-dimensional (2D) cell cultures or animal models [2].

Animal models afford robust platforms for investigating complex inter-organ interactions, particularly in systemic diseases, such as allergies and other non-infectious conditions, that involve multi-etiological factors [3]. However, the utilization of animal models is limited by substantial interspecies variations in gene expression and environmental influences, which restrict their ability to emulate human pathophysiology [4]. Notably, the regulation of gene expression, immune responses, and metabolic processes often diverges markedly between human and animal tissues [5,6]. To address these gaps, there is a pressing need for human-derived in vitro models, such as organoids or engineered tissues, that better replicate physiological and disease processes [7,8]. Organoids replicate vital processes of embryonic development and perform several organ-specific functions [9].

The advent of human pluripotent stem cells, particularly human embryonic stem cells (hESCs) and human-induced pluripotent stem cells (hiPSCs), has revolutionized research on human organ development and disease mechanisms [10,11]. A key advantage of hiPSCs lies in their ability to retain the genetic profile of the donor, making them a powerful in vitro model for examining critical cell–cell interactions and inter-organ communication under disease conditions [12,13]. Moreover, hiPSCs can be directed to differentiate into specific cell types, enabling the study of organ-level interactions using 2D co-culture systems [14]. The ongoing progress in biotechnology and tissue engineering has led to the development of advanced 3D organoid systems, including multilineage organoids, assembloids, and organoids-on-a-chip, that more accurately replicate the architecture and function of human tissues [15,16]. These innovations hold significant promise for generating complex, functional organoids from hiPSCs, enabling the investigation of inter-organ communication during development and disease [17]. In addition, cutting-edge omics tools, such as single-cell and spatial transcriptomics, allow for more detailed analysis of these diverse and intricate cellular systems [18,19].

In recent years, a diverse array of these tissue models has been developed, including organoids that represent the digestive organs [20], kidney [21], lung [22], brain [23], and liver [24].

Organoid models are beneficial for investigating the impacts of genetic differences or environmental factors on organ development, offering insights into the pathophysiological mechanisms of human diseases [7]. They are increasingly used in various research areas, including drug discovery, disease modeling, and personalized medicine [25].

A critical component of organoid development and function is multilineage communication, which refers to the dynamic interactions among various differentiated cell types within the organoid microenvironment [26]. These interactions orchestrate tissue patterning, maintain homeostasis, and drive functional maturation, closely mirroring the complex intercellular signaling found in native organs. The assimilation of multiple cell lineages, including epithelial, stromal, endothelial, and immune cells, enhances the physiological relevance of organoids, particularly for modeling disease processes that involve dynamic crosstalk between diverse cell types such as fibrosis, cancer, and inflammatory disorders [27,28]. The 2D differentiation methods, which begin with pluripotent stem cells, often fail to replicate the complex cellular interactions necessary for organ formation. In contrast, 3D organoid systems can generate more intricate, organ-mimicking tissue architectures [2]. However, the influence of diverse cell-type interactions (heterotypic interactions) on lineage specification remains insufficiently understood.

Physiological “normoxia” varies among embryonic and adult cell types, typically ranging from 2–9% O_2_ (14.4–64.8 mm Hg), compared with 21% O_2_ in the external atmosphere [29,30]. Specific tissues, such as the thymus, kidney medulla, and bone marrow, can function at ≤1% O_2_ (7.2 mm Hg) owing to unique vascular architectures. In adult stem cell niches, which are typically located away from direct vascular supply, oxygen levels can drop to around 1% (7.2 mm Hg) [31,32,33]. One critical aspect of organoid development and function is the role of hypoxia, a condition of low oxygen levels. In organoids, hypoxia can influence cell differentiation, metabolism, and signaling pathways, affecting their overall development and function [34]. Hypoxia is a common feature of many diseases, including cancer, where it can promote tumor growth, invasion, and metastasis [35]. In obesity, adipocytes can grow to 150–200 μm in diameter, surpassing the ~100 μm oxygen diffusion range from nearby vessels, resulting in cellular hypoxia [36,37]. Low oxygen levels, or hypoxia, are not only characteristic of pathological states, such as ischemia, inflammation, and solid tumors [38], but are also present under physiological conditions, including during embryonic development and within certain adult tissues. These low-oxygen environments, often referred to as hypoxic niches, are crucial for guiding cellular differentiation [39,40]. Notably, stem cells typically inhabit these specialized anatomical regions, which play a vital role in regulating their function during development, as well as in tissue repair and maintenance.

Another critical factor in organoid biology is multilineage communication, the intricate interplay between different cell types within the organoid [41,42]. This communication is essential for maintaining tissue homeostasis, regulating cell differentiation, and coordinating responses to external stimuli. In disease models, the disruption of multilineage communication can contribute to disease progression and impact treatment responses.

The link between oxygen availability and mammalian embryogenesis was first recognized in the 1970s, when Morriss and New showed that neural fold formation in ex utero mouse embryos required low oxygen conditions to proceed normally [43]. Subsequent research has revealed the underlying molecular pathways involved in oxygen-mediated developmental regulation, particularly following the identification and characterization of hypoxia-inducible factors (HIFs), dimeric transcription factors that orchestrate cellular responses to reduced oxygen availability [44,45].

Studies using genetic models across various species have further emphasized the critical role of HIFs in development, as embryos lacking key HIF components frequently exhibit multiple developmental abnormalities [46,47]. These findings underscore the role of oxygen as a central regulator of embryogenesis. In this review, we examine how oxygen tension and HIFs influence both embryonic development and stem cell function, while also addressing alternative, HIF-independent pathways, such as the mTOR signaling axis, that contribute to hypoxia adaptation and embryonic growth.

**Figure 1:**
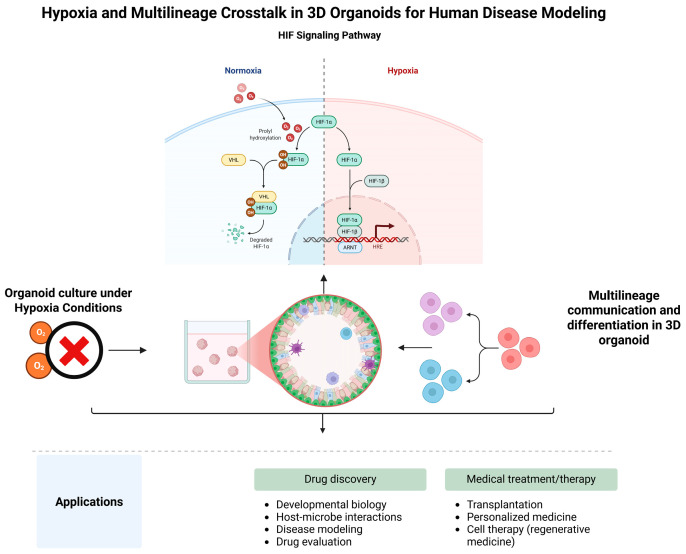
Hypoxia and multilineage crosstalk in 3D organoids increase the HIF signaling pathway. Created with BioRender.com.

This review article further explores the interplay between hypoxia and multilineage communication within 3D organoids and disease models. We discussed how hypoxia modulates organoid development and functionality, the role of multilineage interactions in maintaining tissue homeostasis and modeling disease, and how the convergence of these two factors shapes organoid biology, as presented in Figure 1.

## 2. Hypoxia Synopsis

### 2.1. Background

Oxygen is vital for most eukaryotes, serving to neutralize reactive by-products of mitochondrial respiration [48]. Under low oxygen, cells shift from oxidative phosphorylation to anaerobic glycolysis, enhancing glucose uptake and inducing stress-response proteins that influence survival or apoptosis [49].

Hypoxia describes a state in which tissues experience insufficient oxygen supply, disrupting the homeostatic balance essential for cellular function. It may arise from reduced blood flow, diminished hemoglobin levels, or decreased blood oxygen content. Oxygen deficiency can be localized to specific tissues or systemic, affecting the entire body.

Hypoxia-responsive gene expression is primarily mediated by HIFs or nuclear factor-κB (NF-κB). Three HIF-α isoforms (HIF-1α, HIF-2α, HIF-3α) [50] form functional heterodimers with the constitutively expressed HIF-1β [51]. HIF-1α is present in nearly all human tissues, whereas HIF-2α and HIF-3α show more restricted expression, mainly in specific tissues and developmental stages such as the fetal lung and vascular endothelium [52,53,54].

HIF-α remains stable in hypoxia; however, under normoxia, HIF-1α is hydroxylated at proline residues 402 and 564 within its oxygen-dependent degradation (ODD) domain by prolyl hydroxylases (PHDs) [55,56]. This modification promotes binding to the von Hippel–Lindau (VHL) E3 ligase, leading to ubiquitination and proteasomal degradation [57,58,59]. HIF-1α activity is also regulated by hydroxylation of Asn803 in its C-terminal transcriptional activation domain (C-TAD). Under normoxia, factor inhibiting HIF-1 (FIH-1) catalyzes this modification, preventing HIF-1α from interacting with the coactivators CBP/p300 [60,61,62].

**Figure 2:**
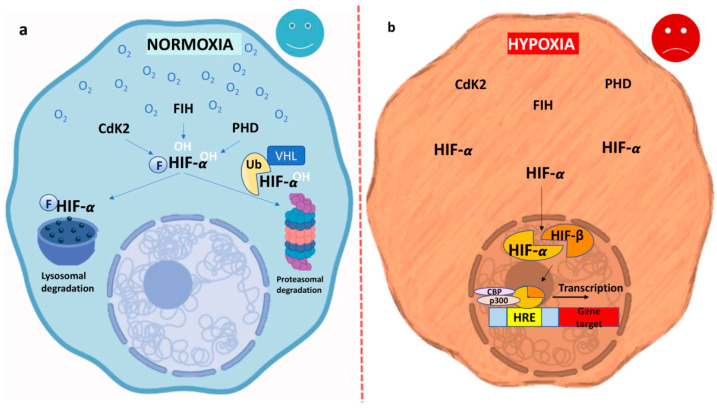
HIF-α regulation in normoxia and hypoxia. (**a**) In normoxia, HIF-α is hydroxylated at Pro402 and Pro564 in the oxygen-dependent degradation (ODD) domain by prolyl hydroxylases (PHDs) and at an asparagine residue by factor inhibiting HIF-1 (FIH-1). These modifications promote binding to the Von Hippel–Lindau (VHL) E3 ligase, leading to ubiquitination, proteasomal degradation, and suppression of hypoxia-responsive gene transcription. (**b**) Under hypoxia, reduced oxygen and hydroxylation cofactors limit PHD and FIH activity, preventing HIF-α hydroxylation. Stabilized HIF-α accumulates, translocates to the nucleus, and dimerizes with HIF-β. The HIF-α/β complex binds to hypoxia-response elements (HREs), activating genes, such as EPO, IGF-2, TGF-α, VEGF, MMP2, and α/β integrins, as well as those involved in glucose metabolism. This figure is reproduced from [68]. © The Author(s) 2022. This article is licensed under a Creative Commons Attribution 4.0 International License.

Under hypoxia, limited oxygen and hydroxylation cofactors reduce HIF-1α hydroxylation [63,64]. As a result, HIF-1α accumulates, translocates to the nucleus, and dimerizes with HIF-1β. These complex binds hypoxia-response elements in oxygen-regulated genes, modulating their expression [65,66,67]. A schematic diagram of the oxygen-dependent regulation of HIF-α is presented in Figure 2, summarizing its post-translational degradation under normoxia and stabilization-mediated transcriptional activation during hypoxia.

Hypoxia may develop abruptly or progress into a chronic condition. Prolonged oxygen deprivation triggers adaptive mechanisms, such as enhanced erythropoiesis, to increase oxygen transport, angiogenesis to improve blood supply, and metabolic adjustments to lower oxygen consumption and preserve redox balance under sustained low-oxygen conditions [69].

### 2.2. Hypoxia Physiological Applications

#### 2.2.1. Physiological Hypoxia in Immune Cell Environments

The bone marrow, localized in the central cavity of bones, is the primary site of adult hematopoiesis, generating all immune cell types from pluripotent, self-renewing hematopoietic stem cells (HSCs) [70]. Under normal conditions, the marrow is hypoxic [71], a state that supports HSC homeostasis by regulating quiescence, metabolism, survival, proliferation, and differentiation via hypoxia-sensitive transcription factors, particularly HIFs, and through soluble factors from other niche cells such as adipocytes and endothelial cells [72].

Hypoxia, or pharmacological hydroxylase inhibition, enhances HSC quiescence in an HIF-dependent manner [73,74], likely through metabolic reprogramming that favors glycolysis over oxidative phosphorylation [75,76,77,78,79]. Loss of HIF1β impairs HSC viability through the disruption of HIF signaling [80], although the precise role of HIFs remains debated, with some evidence suggesting that HIF2α is dispensable for HSC maintenance [81]. Overall, physiological hypoxia in bone marrow appears to regulate HSC viability and function, mainly through the HIF pathway.

#### 2.2.2. Role of Hypoxia During Development

The impact of oxygen has been reported during embryonic development and organogenesis. Deletion of HIF-1α in lung epithelium using an SP-C promoter–driven Cre led to impaired epithelial maturation and a respiratory distress syndrome–like phenotype [82]. Similar results were previously demonstrated in HIF-2α knockout mice, where VEGF treatment ameliorated the defect [83]. Cardiomyocyte-specific deletion of HIF-1α results in embryonic lethality at approximately E11–E12, accompanied by impaired cardiac looping, a phenotype similar to that seen in global HIF-1α knockout mice [84,85]. Neural crest cells originate from the neural plate border and, at neural tube closure, encounter epithelial-to-mesenchymal transition (EMT) before migrating throughout the embryo to contribute to structures such as the peripheral nervous system, craniofacial mesectoderm, and melanocytes [86,87]. Studies have linked hypoxia and HIF signaling to neural crest EMT, migration, and peripheral nervous system development [88,89,90].

The role of hypoxia in orchestrating multilineage communication is central to organoid maturation, as it directly influences vascularization and tissue complexity. This underscores the necessity of developing advanced organoids that integrate parenchymal, mesenchymal, immune, and vascular components to achieve physiologically mature and fully functional tissues in vitro [41]. Pathological hypoxia is a condition where oxygen deprivation harms body tissues [91]. It can be seen in postnatal conditions, which can obstruct several functions [92], aiding in tumor growth or vasculature dysfunction [93]. Nonetheless, prenatally, hypoxia is a crucial physiological signal that contributes to organ and blood vessel formation during early embryogenesis [29,94,95]. The hypoxic environment has a wide range of various impacts on different organs in different species, depending on the fetal stage, as shown in Table 1 [96].

In embryonic development during organogenesis, oxygen levels vary among developing organs such as maternal and fetal blood mix at mid-gestation [94,95]. During organogenesis, embryos are most dependent on glycolysis as the source of energy [95].

This process is accompanied by HIFs, which regulate various genes influencing organ function. Notably, although the placenta’s primary role is to transport oxygenated blood, it develops under hypoxic conditions [85]. HIF expression in the mouse embryonic placenta is crucial for sustaining chorioallantoic interactions between the allantois and chorion. It enhances MAP kinase signaling to promote vascular branching and prevents premature formation of syncytiotrophoblasts [94]. The cardiovascular system (CVS) shows a physiological hypoxia phenomenon where different parts express different levels of hypoxia during various stages of embryogenesis [14]. Especially since CVS plays an important role in oxygen delivery through nascent vasculature [14]. The mouse heart is also developed under hypoxic conditions to guarantee healthy cardiac morphogenesis [85,97]. However, as heart development progresses toward the later stages of embryogenesis, the hypoxic conditions are limited to the myocardium only [85,96]. In rats, upon initiation of a heartbeat, HIF-α, along with its downstream targets, was upregulated in the heart primordium [98]. The high vascularization and cell density in the mice bones led to hypoxia, impacting the limb length [99]. Physiological hypoxia varies greatly within different brain regions [13]. The developing central nervous system (CNS) and kidney in mice are controlled by embryonic neural erythropoietin-producing cells in regions where HIFs are expressed, aiding in maintaining erythropoietin production [100]. Moreover, glycolysis and hypoxia help in the neural tube formation of mice [101]. The HIFs expression in the CNS is prolonged; it starts early in the neuroepithelium and might be extended until birth [102]. HIF-α took a turn in the neural tube closer in both mice and rats [101,103]. The prolonged hypoxic episode during the late phases of human organogenesis might lead to several complications, including limb deformities and tissue loss, cell loss of the developing brain, cleft lip, and hypospadias [95].

**Table 1:** Role of HIFs in the development of different organs in animal models.

Animal Model	Organ	Developmental Stage	Role of HIFs	References
Mice	Placenta	E3.5−14.5	Growth and vascularization	[94,104]
Mice	CVS	E7.75−15	Cardiac morphogenesis, vascular endothelial development, and endocardial cushion development	[85,96,97,105,106]
Mice	Bone	E10−18.5	Limb length, chondrogenesis, and osteogenesis	[99,107]
Mice	CNS	E7.5−E11.5	Erythropoiesis, neural tube formation/closure	[100,101,102,108]
Wistar rats	Heart	Starting at E10	Cover high energy demand after heartbeat initiation	[98]
Rats	CNS	GD11	Neural tube closure	[103]
Rats	Ears	GD11	Otic vesicle closure	[103]
Quail	Myocardium	E4−6	Ventricular myocardium trabecularization	[109]

Abbreviations: CVS: Cardiovascular system; E: Embryonic day; CNS: Central nervous system; GD: Gestational day.

#### 2.2.3. Hypoxia-Related Tissue Regeneration

Several experimental studies show that systemic hypoxia can enhance tissue regeneration [110,111,112,113]. Hypoxia-mediated angiogenesis is essential for tissue repair and regeneration [114]. Acute hypoxia can promote wound healing through HIF pathways; however, timely restoration of normal oxygen is essential, as sustained hypoxia hinders recovery [115]. During the inflammatory phase, oxygen supports ATP-dependent processes; after hemostasis, hypoxia triggers early healing responses through HIF regulation [116].

Hypoxia leads to ROS production, activating platelets and monocytes to release cytokines and growth factors, including TGF-β1, VEGF-A, and TNF-α [117]. These mediators recruit neutrophils and macrophages to wound and activate fibroblasts [117,118]. TGF-β1 promotes procollagen gene transcription, enhancing fibroblast migration, with acute hypoxia increasing its mRNA expression, collagen synthesis, and fibroblast proliferation [119,120].

Hypoxia-induced HIF-1α enhances peripheral axon regeneration, while its knockdown or knockout impairs sensory axon repair. VEGF-A, an HIF-1α target, is upregulated in injured neurons, promoting axon growth in sensory and motor neurons and accelerating neuromuscular junction reinnervation. These findings identify HIF-1α as a key transcriptional regulator of neuronal and axonal regeneration [121]. Preclinical studies demonstrated that intermittent hypobaric hypoxia (IHHP) in rats enhances neural regeneration and repair while reducing ischemia–reperfusion-induced neurological deficits, indicating potential clinical utility [122].

In mice, one week of hypoxia after myocardial infarction reduced oxidative metabolism, ROS production, and DNA damage, while reactivating cardiomyocyte mitosis [123]. This treatment decreased fibrosis, improved ventricular function, and promoted regeneration from preexisting cardiomyocytes, indicating that gradual hypoxemia can restore the adult heart’s endogenous repair capacity. In rats, drug-induced hypoxia accelerated liver regeneration comparable to that seen with portal vein ligation and parenchymal transection [124]. Hypoxia also exerts hepatoprotective effects via HIF-2α–mediated macrophage reprogramming [125]. In hepatic stellate cells, hypoxic stimulation induces VEGF-A–driven proliferation of hepatic sinusoidal endothelial cells, and in vivo models show that an elevated von Willebrand factor accompanies rapid regeneration [126].

#### 2.2.4. Hypoxia-Mediated Growth Factors Modulation

The primary mediator of the cellular response to hypoxia is the HIF family of transcription factors, particularly HIF-1α and HIF-1β. Under hypoxic conditions, these HIF-α subunits escape proteasomal degradation, translocate to the nucleus, and heterodimerize with HIF-β, binding to hypoxia-response elements in the promoters of target genes [127]. This transcriptional activation leads to the upregulation of numerous growth factors, including vascular endothelial growth factors (VEGF), FGFs, platelet-derived growth factors (PDGF), and transforming growth factor-beta (TGF-β). VEGF, a potent angiogenic factor, is particularly highly induced by hypoxia, promoting neovascularization to restore oxygen supply. FGFs contribute to cell proliferation, migration, and angiogenesis, while PDGFs are involved in pericyte recruitment and ECM remodeling. TGF-β, known for its multifaceted roles, can promote angiogenesis, epithelial–mesenchymal transition (EMT), and immunosuppression under hypoxic conditions. The coordinated upregulation of these growth factors under hypoxia facilitates adaptive responses, such as erythropoiesis and angiogenesis, but also drives detrimental processes, including tumor growth, metastasis, and fibrosis [128,129,130,131].

#### 2.2.5. Hypoxia in the Stem Cell Niche

**Figure 3:**
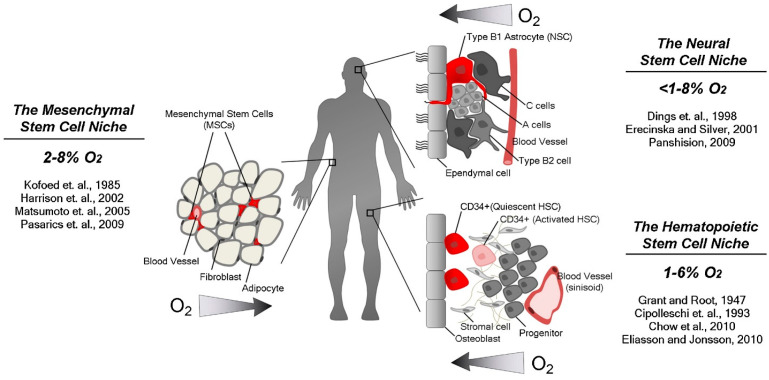
This figure depicts the oxygen tension in several stem cells with related references. The figure is reproduced from [33] with permission. Copyright © 2010 Elsevier Inc. All rights reserved.

As an attempt to maintain a stem cell niche that is known to have low partial oxygen pressure (PO_2_) [132], saving stem cells against DNA damage and oxidative stress, hypoxia plays a role in setting stem cells into dormancy, forcing the respiration mode shift towards anaerobic glycolysis [32,132,133]. Supporting such a niche under hypoxic conditions helps to maintain stem cell potency and migration capacity [132]. As previously discussed, human embryonic cells tend to differentiate and develop under hypoxic conditions. Ezashi et al. reported that hESCs tend to grow best at conditions where oxygen is between 3–21%. Decreasing the oxygen levels below 1% has no impact on the hESC potency but does have an effect on their proliferation [134]. Different stem cell niches with oxygen levels are presented in Figure 3. Adelman reported a similar effect in murine placental trophoblast stem cells, where the stem cells differentiated into a more spongiotrophoblast cell fate in hypoxic conditions [135]. Studies over the past century revealed that HSCs are dormant in a hypoxic region of the bone marrow known as the endosteal niche [74,136,137]. Shedding light on the role of HIF-1α protein in stem cells, the same study reported that the absence of HIF-1α protein results in cell-cycle quiescence loss [74]. While MSCs are usually present in regions with high blood supply, they still maintain low oxygen levels within their niche.

### 2.3. Pathological Hypoxia

#### 2.3.1. Inflammatory Diseases

In inflammation, hypoxia, often driven by neutrophil oxidative bursts, triggers hypoxia-dependent transcriptional programs in various cell types, influencing metabolism, cytokine production, differentiation, and survival at the inflammatory site [138]. Acute inflammation is marked by abundant neutrophil infiltration and major metabolic changes [139]. Elevated oxygen consumption in inflamed tissues that is driven by infiltrating immune cells, local proliferation, and oxygenase activity creates “inflammatory hypoxia”, a pathological immune niche [139].

Hypoxia effects vary by tissue; in the lung, it prolongs neutrophil survival, promoting injury through the release of antimicrobial factors toxic to epithelial cells [140]. Activation of AMPK appears protective, as metformin reduced neutrophil accumulation, dampened HIF-1 signaling, and preserved epithelial integrity in a murine sepsis-induced lung injury model [141].

In chronic inflammation, hypoxia can be either pro- or anti-inflammatory, depending on the cell type. In inflammatory bowel disease (IBD), intestinal mucosal hypoxia intensifies due to elevated metabolic demands and inflammation-induced vascular dysfunction, contributing to fibrosis [142]. In epithelial cells, hypoxia enhances barrier protection via HIF-regulated genes [143], whereas in immune cells, it can boost survival and activity through HIF activation. Overall, pharmacological HIF activation via hydroxylafse inhibition has shown strong anti-inflammatory effects [138]. Patients with chronic granulomatous disease, caused by defective NADPH oxidase in neutrophils, often develop IBD-like symptoms [144].

HIF regulates several molecules that protect the intestinal barrier in IBD, including claudin-1 (CLDN1), essential for tight junctions [145], and creatine kinase enzymes in adherens junctions are expressed in an HIF-2α–dependent manner [146]. Other HIF-dependent components include mucin MUC-3, intestinal trefoil factor [147,148], and the antimicrobial peptide human β-defensin 1 (hBD1), whose activity is enhanced under hypoxia [149].

Clinically, intestinal epithelial cells in Crohn’s disease (CD) and ulcerative colitis (UC) show elevated HIF-1α and HIF-2α levels [150], with UC biopsies revealing a correlation between HIF expression and disease severity in both active and remission stages [151].

#### 2.3.2. Infection

Pathogen-infected tissues often become hypoxic and show elevated HIF activity [152,153], driven by bacterial oxygen consumption, biofilm formation, and inflammation-related hypoxia [153]. In cystic fibrosis, Pseudomonas aeruginosa commonly inhabits such low-oxygen niches [154], where hypoxia can enhance immune cell survival and function [155] but also influence bacterial behavior, reducing virulence, promoting drug resistance, and supporting chronic infection [156,157]. Bacterial hydroxylases may further modulate P. aeruginosa virulence under hypoxia [158]. Thus, infection-site hypoxia shapes both host immunity and pathogen activity, impacting disease progression.

#### 2.3.3. Pulmonary Diseases

Fetal lung hypoxia can result from reduced uterine blood flow (e.g., placental insufficiency, maternal smoking) or maternal oxygen deprivation (e.g., high altitude). Such hypoxia may cause pulmonary hypertension, increased airway resistance, and suppressed fetal breathing [159]. Infants of smoking mothers often have smaller lungs with impaired function for up to 18 months [160]; in sudden infant death syndrome (SIDS) cases linked to heavy maternal smoking, airway walls were thickened and lumens were narrowed [161].

In adults, hypoxia activates pulmonary endothelial responses that adjust blood flow and pressure but may also cause endothelial damage, increasing vascular permeability, smooth muscle tone, cell proliferation, and thrombosis risk [162]. Acute hypoxia reversibly constricts pulmonary arterial smooth muscle cells (PASMCs) by inhibiting voltage-gated K^+^ channels [163], producing a biphasic pressure-response initial contraction with vasorelaxation, followed by sustained constriction characteristic of hypoxic pulmonary vasoconstriction (HPV) [164].

Chronic hypoxia can lead to pulmonary hypertension (PH), defined as mean pulmonary arterial pressure >25 mm Hg at rest or >30 mm Hg during exercise [165,166], and is linked to lung diseases such as sleep apnea and chronic obstructive pulmonary disease (COPD) [162]. Endothelial dysfunction in PH suggests that vascular remodeling may involve abnormal angiogenesis, similar to tumor growth, with HIF-1α as a key regulator [167,168].

In hypoxic pulmonary vascular cells, reduced mitochondrial ROS inhibits oxygen-sensitive channels, causing depolarization, calcium influx, and HPV [169]. Mitochondrial damage can produce false hypoxic signals, chronically activating HIF-1α—even in normoxia—downregulating oxygen-sensitive channels, and driving persistent vasoconstriction, apoptosis resistance, and mitochondrial hyperpolarization, linking PH to other pathologies [170].

#### 2.3.4. Hepatic Diseases

Acute and chronic liver diseases, including hepatitis, portal hypertension, and fatty liver disease linked to obesity or alcohol abuse, can progress to steatosis, steatohepatitis, fibrosis, cirrhosis, and hepatocellular carcinoma.

In non-alcoholic fatty liver disease, hypoxia may aggravate the disease pathogenicity through HIF-2α–mediated lipid metabolism changes [171]. Additionally, a recently identified HIF-1α/PTEN/NF-κBp65 pathway promotes fibrosis via PTEN/p65, suggesting a potential therapeutic target [172]. Hypoxia may exacerbate chronic liver injury, while HIF-2α can reprogram hepatic macrophages to protect against acute damage [125]. Brief ischemic episodes, known as ischemic preconditioning, can reduce necrosis and apoptosis in liver ischemia–reperfusion injury, offering protection during liver surgery and transplantation [173].

#### 2.3.5. Renal Diseases

Renal tubules have limited anaerobic capacity and high oxygen consumption [174], making the kidney especially vulnerable to ischemia. Hypoxia promotes macrophage accumulation in renal tissue, triggering profibrotic cytokine release and activating fibroblasts, which also respond directly to hypoxia by increasing ECM deposition [175]. Along with inflammatory cell recruitment and tubular epithelial injury, this drives tubulointerstitial fibrosis, exacerbating hypoxia and progressing to chronic kidney disease. Elevated endothelin expression further exacerbates fibrosis through vasoconstriction [133,154].

Factors, such as anemia, hypertension, and kidney injury, can further exacerbate renal hypoxia [176]. Acute kidney injury (AKI) is a major cause of renal hypoxia, which persists beyond the acute phase, leading to prolonged oxygen deprivation. This status downregulates the proangiogenic VEGF-A isoform 164 while upregulating dysangiogenic isoforms 120 and 188 [177], causing loss and narrowing of renal capillaries [178]. The initial hypoxic insult alters vascular architecture, driving chronic hypoxia, which damages tubular epithelial cells and induces apoptosis. Hypoxia also modulates gene expression via HIF-1, which binds the CD18 promoter, regulating β2 integrin subunit expression through chromatin remodeling and histone modification [179,180].

#### 2.3.6. Heart Diseases

The heart is highly sensitive to oxygen fluctuations due to oxygen’s role in nitric oxide (NO) production, which regulates cardiac and vascular contractility via oxygen-dependent S-nitrosylation of cysteine residues [181]. This process influences excitation–contraction coupling, calcium flow, homeostasis, and injury response [181,182]. Oxygen also drives ROS generation, which can promote myofibroblast differentiation but may also induce cell death through mitochondrial damage [183,184].

Elevated NADPH oxidase activity promotes atherosclerosis by producing superoxides that oxidize LDL, causing endothelial dysfunction and foam cell formation [185]. HIF influences various cardiac phenotypes, with its stabilization often providing cardioprotection via ischemic preconditioning, brief, non-lethal hypoxia cycles with reperfusion that reduce myocardial injury [176]. In contrast, chronic hypoxia impairs calcium handling and cardiac metabolism, potentially resulting in heart failure [186].

## 3. Overview of Organoids Technology

Organoid technology represents a transformative advancement in biomedical research, enabling the formation of miniature, 3D organ-like structures from stem cells or tissue fragments in vitro [187]. These “mini-organs” mimic the architecture, cellular heterogeneity, and functional characteristics of their in vivo counterparts, thereby bridging the gap between traditional 2D cell cultures and animal models [188].

Organoids can be derived from various sources, including embryonic stem cells, induced pluripotent stem cells, adult stem cells, cancer cells, primary tissues, xenografts, and even mature cells [189,190,191]. Cancer-derived organoids, or tumoroids, are generated by dissociating tumor tissue into single cells or small clusters and embedding them in an extracellular matrix (e.g., Matrigel or defined hydrogel) under conditions that preserve tumor heterogeneity [192,193,194]. They replicate the architecture, histopathology, genetic and mutational profiles, and therapeutic responses of the original tumor. ECM is essential, as it provides structural support and biochemical signals that direct cellular self-organization, proliferation, and differentiation. Growth factors added to the culture media, such as EGF for epithelial organoids and fibroblast growth factor 10 (FGF10) for hepatic and gastric tumoroids, further guide organ-specific development [188]. The generation of hiPSC-derived organoids typically follows a stepwise differentiation process, guiding hPSCs into lineage-specific progenitors using stage-appropriate morphogens and growth factors that mimic developmental signaling [195]. These protocols aim to replicate human organogenesis, resulting in organoids that often resemble immature or fetal tissue rather than fully developed adult tissue, as seen in patient-derived organoids [196,197]. A defective differentiation strategy can lead to the presence of unintended cell types [198,199,200]. To address this, researchers benchmark hiPSC-derived organoids against fetal tissue, using it as an in vivo reference to refine developmental accuracy and cellular composition [42,198,201].

hPSC-derived organoids represent a rapidly advancing area of research, with broad applications in disease modeling and drug discovery. Owing to their ability to differentiate into virtually any cell type, hPSCs enable the generation of diverse organoid systems. Vandana et al. reviewed a wide range of these models, encompassing endoderm-derived organoids (lung, liver, intestine, pancreas), mesoderm-derived organoids (heart, kidney, skeletal muscle, blood vessels), and ectoderm-derived organoids (neural, retinal) [202]. This enables access to cell types that were previously challenging to isolate and culture, including the generation of complex structures such as brain and cardiac organoids [203]. Patient-derived cells can be reprogrammed into hPSC-derived organoids using specialized differentiation media and growth factors. These organoids serve as versatile platforms for drug screening, immune response studies [202,203] and investigations into genetic disorders and infectious diseases [204].

Organoids offer significant advantages. They recapitulate organ-specific microanatomy and cell interactions that are absent in monolayer cultures, thus providing high physiological relevance [205]. Furthermore, organoids retain the genetic, morphological, and functional characteristics of their tissue of origin, which makes them powerful models for studying development, disease mechanisms, and personalized therapies [206]. Organoids are highly versatile, having been established for a wide array of tissues, including the intestine, liver, brain, kidney, and tumors, and can be propagated in the long term or cryopreserved [7]. Genetic manipulation tools, such as CRISPR/Cas9, have been effectively used in organoid systems, enhancing disease modeling by enabling precise gene editing and simulation of disease progression [189,207].

Applications of organoids bridge diverse research domains, including developmental biology, regenerative medicine, toxicology, infectious diseases, and high-throughput drug screening [208,209]. Organoids also present notable advantages, including compatibility with comprehensive omics analyses, a small size that enables high-resolution 3D and even 4D imaging, and the capacity to generate large datasets suitable for training artificial intelligence models [210]. On the other hand, the use of organoids in high-throughput screening is hindered by some limitations. First, although their small size aids in high-quality images and precise machine learning assessment, their small size interferes with their ability to be scaled for in vivo or clinical transplant [211]. Second, organoids are static, which contradicts the dynamic nature of humans, as organoids lack blood vessels, immune cells, and microbes; their absence affects cell-to-cell interaction and scalability [212]. Moreover, in complex organs, like the brain, organoids might lack heterogeneity and reproducibility [213]. Third, the variation in organoid size, morphology, and maturation across batches disrupts assay consistency and hampers automation, while manual handling requirements and dependence on extracellular matrices, such as Matrigel, pose additional obstacles to scalable, fully automated workflows. Finally, high-content imaging generates complex datasets requiring advanced computational analysis; slow-growing or rare organoid types further restrict throughput, making rapid, large-scale screening challenging [214].

Patient-derived tumor organoids are increasingly used to investigate cancer heterogeneity and predict drug response, while kidney organoids serve as models for nephrotoxicity and congenital disorders [215]. Integration with organoid-on-a-chip platforms and microfluidics enables researchers to simulate inter-organ interactions and improve physiological relevance, thus moving closer to complex disease modeling and personalized therapeutics [216,217].

Recent innovations include the use of biomaterial scaffolds and microfluidic systems to mimic the physical and biochemical environment of native tissues, as well as the incorporation of single-cell sequencing and artificial intelligence for deeper phenotypic analysis and predictive modeling [217,218,219]. Despite these advances, organoid research still faces challenges in scalability, reproducibility, vascularization, and clinical standardization areas that continue to be intensively explored. Integrating organoid technology with tissue engineering has led to the creation of organoids-on-a-chip using microfluidic platforms [220]. These systems enable the construction of advanced, tissue-integrated biomimetic organoids that closely replicate human organ function and disease progression in vitro [221]. These humanoid organoid platforms facilitate both macroscopic and microscopic investigations of intercellular signaling and communication across tissues, paving the way for multi-organ models that faithfully replicate complex inter-organ interactions. These systems are proving valuable for studies in organogenesis, drug toxicity testing, and therapeutic efficacy evaluation [222].

## 4. Multilineage Communication

### 4.1. Overview

Although hypoxia may directly affect only certain cell types, the overall response occurs at the tissue level through multilineage interactions, particularly between stromal elements (such as endothelial and mesenchymal cells) and tissue-specific epithelial cells. These epithelial–mesenchymal communications often drive organ maturation.

In lung regeneration models, for example, VEGF signaling stimulates endothelial expression of MMP14 [223], which is further enhanced by FGF2/FGF5 from basal epithelial cells [224]. MMP14 then releases EGF-like domains that activate epithelial progenitors, expanding their pool and supporting alveolar repair. Similarly, the diminution of Matrix GLA Protein in lung endothelial cells during development disrupts the signaling balance, allowing BMP4 to induce VEGF and VEGFR1 expression, which leads to endothelial HGF production and aberrant hepatic differentiation of the pulmonary epithelium [225]. Pericytes are key mediators of vascular–epithelial signaling during alveolar development, secreting angiopoietin to support endothelial angiogenesis and integrity, as well as HGF to stimulate epithelial-driven alveologenesis [226].

Organ development relies on intricate interactions among various embryonic tissues. The 3D culture systems have emerged as powerful platforms for generating tissue constructs that closely mimic the functional characteristics of native organs [227]. Organoids preserve the genetic profile of donors, making them highly valuable for disease modeling, investigating biological mechanisms underlying pathology, and exploring developmental processes [228]. For example, in the cardiovascular field, organoid research has expanded to encompass myocardium, vascular structures, and cardiac valves, which offer versatile models for studying cardiovascular diseases [229].

hiPSCs provide a versatile platform for disease modeling, offering unprecedented opportunities to dissect the mechanisms underlying human biology and physiology. They have been widely applied to investigate interactions across multiple cell types and systemic levels [17]. A 2D co-culture system using hiPSCs and their derivatives provides a minimal yet practical format for modeling multilineage interactions in vitro [17]. The first hiPSCs were reportedly cultured in a 2D co-culture system with irradiated mouse embryonic fibroblasts. They demonstrated therapeutic efficacy in mouse models of acute kidney injury caused by ischemia/reperfusion. Therefore, maintaining multipotency in hiPSC-derived progenitors often still relies on the use of feeder cells [230,231].

Taken together, the crosstalk between these lineages is crucial for orchestrating organ development and repair. Advances in 3D culture have enabled the recreation of such complex multilineage interactions in organoid systems, which faithfully capture native tissue architecture, genetic background, and functional behavior.

### 4.2. Multilineage Communication, Cellular Models, and Applications

**Figure 4:**
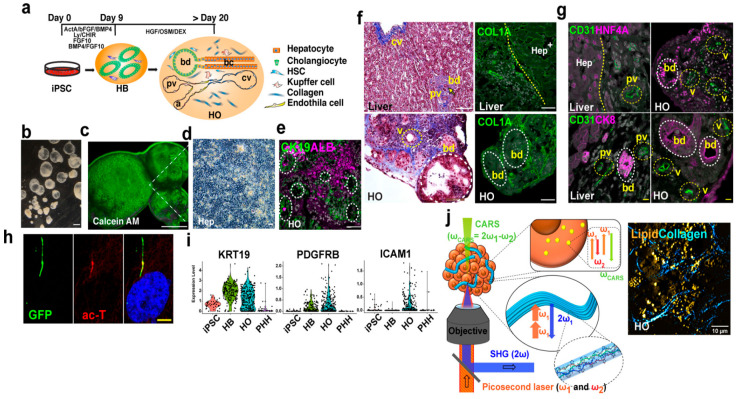
Development of human multilineage hepatic organoid model: (**a**) schematic of the in vitro system used to guide iPSCs toward hepatic organoid (HO) differentiation, highlighting central vein (cv), portal vein (pv), bile duct (bd), bile canaliculus (bc), and artery (a); (**b**) low-magnification bright-field image of day-21 HOs (scale bar = 500 μm); (**c**) calcein AM staining confirms cell viability within organoids (scale bar = 500 μm); (**d**) high-power view from (**c**) shows polygonal hepatocyte-like cells with lipid vesicles; (**e**) immunostaining reveals albumin^+^ hepatocytes and CK19^+^ cholangiocytes. Dotted circles mark bile ducts (scale bar = 50 μm); (**f**) left: trichrome staining shows liver-like structures in HOs. Right: collagen immunostaining highlights peri-ductal and vascular zones (scale bar = 50 μm); (**g**) HO immunostaining for endothelial (CD31) and hepato-biliary (HNF4A, CK8) markers reveals bile ducts, portal veins, and venules (scale bar = 50 μm); (**h**) primary cilium visualized using ARL13B-GFP and acetylated tubulin staining (scale bar = 5 μm); (**i**) scRNA-seq shows expression of multilineage markers (CK19, PDGFRB, ICAM1); and (**j**) schematic of SHG/CARS imaging (left) and merged image (right) of day-21 HOs showing lipid stores (yellow) and collagen fibers (cyan), scale bar = 10 μm. This figure is reproduced from [232]. Copyright © 2021, The Author(s). This is an open access article distributed under the terms of the Creative Commons CC BY license.

Multilineage organoid systems represent a major advancement over traditional single-cell-type models by incorporating diverse populations, such as epithelial, mesenchymal, endothelial, and immune cells, to replicate native tissue complexity [41]. This heterotypic cellular arrangement enables critical paracrine and contact-dependent signaling that drives organoid differentiation, morphogenesis, vascularization, and functional maturation. A human multilineage hepatic organoid model was developed to study liver fibrosis in autosomal recessive polycystic kidney disease (ARPKD) (Figure 4) [232]. iPSCs were differentiated into hepatic organoids using sequential growth factors; the common disease-causing PKHD1 Thr36Met mutation was introduced homozygously into three iPSC lines via CRISPR/Cas9 and piggyBac transposition.

Liver bud organoids composed of hepatic, stromal, and endothelial cells showed developmental-like vasculature formation through VEGF-mediated crosstalk, closely mimicking in vivo architecture. Likewise, multilineage brain and cardiac “assembloids” recapitulated regional patterning and exhibited enhanced maturation and disease-modeling capacity. As a result, such integrated organoid systems offer a powerful platform for studying complex tissue dynamics and for advancing translational applications in disease modeling and regenerative medicine.

iPSC-derived hepatocyte-like cells (iHeps) improved the functional maturation of hiPSC hepatocytes by enabling controlled cell–cell interactions in a 2D culture system. This platform has shown considerable value for drug screening, investigating the molecular pathways of iHep differentiation, modeling liver diseases, and advancing human-on-a-chip technologies for multi-organ response testing [233]. Furthermore, models for synaptic dysregulation in a hiPSC cell model of mental disorders provide new insights into the molecular and synaptic etiopathology of psychiatric disorders [234]. When co-cultured with hiPSC-derived astrocytes, hiPSC-derived neurons demonstrated sensitivity to convulsant compounds, highlighting their promise as a model system for evaluating the risk of drug-induced seizures [235].

The maturation of hiPSC-derived cardiomyocytes can be promoted by soluble mediators secreted by MSCs, which act through receptor-driven signaling pathways. [236]. The 2D culture systems offer significant advantages, being highly scalable and well-suited for rapid investigation of cellular, transcriptional, genetic, and signaling processes. However, they fall short of replicating the physiological complexity inherent in three-dimensional in vitro models [17].

Human elongating multilineage organized gastruloid technology recapitulated critical features of trunk neurodevelopment, including integration between neural and cardiac lineages, demonstrating that the transition from 2D culture to 3D aggregates promotes the coordinated emergence of polarized neural and cardiac domains [237].

hiPSCs cultured within PNIPAM-AA microgel 3D constructs showed superior multilineage differentiation, with significantly elevated mRNA levels of chondrogenic, osteogenic, and adipogenic markers relative to both pellet and 2D cultures [238]. A 3D co-culture system combining mesenchymal stromal cells with differentiated osteoblasts on human bio-derived bone scaffolds effectively supported active multilineage hematopoiesis in vitro. This strategy not only fosters the self-renewal and ex vivo expansion of hematopoietic stem/progenitor cells (HSPCs) but also maintains primitive hematopoietic stem cells (HSCs) with superior phenotypic and functional characteristics [239].

Advances in clinical dentistry have led to the development of sophisticated scaffold designs that can generate precise morphogen gradients. These engineered systems enable the creation of in situ models for investigating tissue differentiation and is promising for the future engineering of multilineage tissues [240]. A 3D bioprinting approach has been established that enables precise spatial organization of glioblastoma constructs, providing a robust platform for preclinical drug sensitivity assays and investigations of the tumor microenvironment (TME). This system revealed that glioma-associated stromal cells exhibit greater resistance to chemotherapeutic agents in 3D-printed tumor models compared to conventional 2D monolayer cultures, more accurately mirroring clinical outcomes [241]. Taken together, findings from both 2D and 3D systems have advanced our understanding of cellular interactions. While 2D co-culture remains a convenient and accessible platform for studying multi-cell-type dynamics, the development of more sophisticated 3D models is essential to achieve higher physiological relevance.

Multilineage organoids provide a robust platform for examining organogenesis and exploring the dynamic interplay between the endoderm and cardiac tissues during morphogenesis, spatial patterning, and tissue maturation [26]. These organoids were capable of mimicking critical developmental processes that occur post heart-tube formation and can sustain functional tissue structures for extended periods of up to 100 days [242]. Moreover, they created a supportive niche that fostered intestinal tissue development while simultaneously enhancing the maturation and functional stability of cardiomyocytes. In comparison to traditional animal models, multilineage organoids provide a more precise and human-relevant perspective on inter-tissue communication. Consequently, they are emerging as valuable tools for building physiologically accurate in vitro models of human embryonic development.

### 4.3. Multilineage Communication Platforms

#### 4.3.1. Assembloids

Organoids are becoming increasingly sophisticated, aiming to emulate the complexity of multiple human tissues more accurately. Nonetheless, current multilineage organoids still fall short in establishing intricate cellular interaction networks and often lack the spatial organization necessary for recreating true tissue microenvironments [243,244]. To overcome these shortcomings, researchers have developed assembloids, composite structures formed by merging multiple organoids [245,246]. Pioneered by Sergiu Pașca’s team, assembloids represent a significant advancement of the 3D multicellular modeling system [247]. These structures are generated by integrating two or more regionally specified organoids along with additional cell or tissue types.

**Figure 5:**
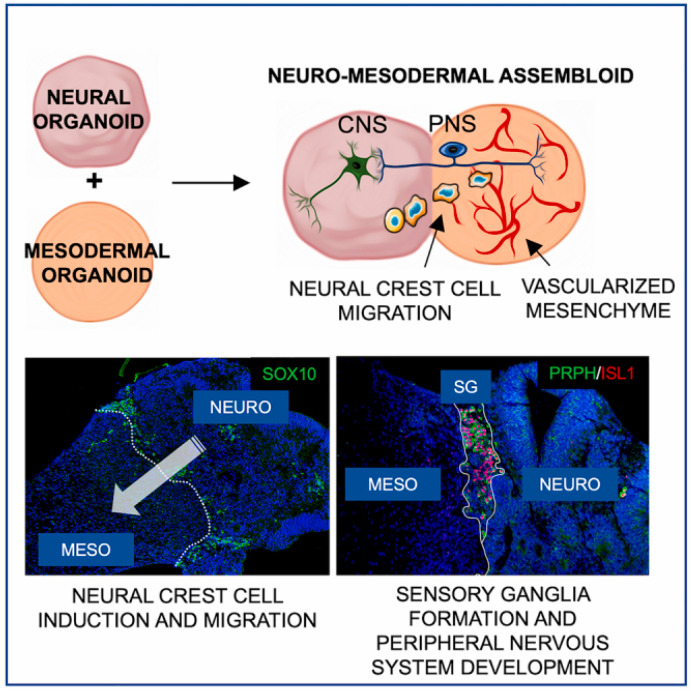
Neuro-mesodermal assembloid model. This figure is reproduced from [248] with permission. © 2023 The Authors. This article is available under the Creative Commons CC-BY-NC-ND license.

Wörsdörfer et al. developed a neuro-mesodermal assembloid that models peripheral nervous system development, including neural crest induction, migration, and ganglion formation (Figure 5) [248]. The formed ganglia extend projections into both neural and mesodermal compartments and interacts with a forming vascular plexus to create a neurovascular niche. Functional sensory activity was confirmed by capsaicin responsiveness.

Assembloids offer a powerful approach to studying complex cellular coordination within and across tissue systems; they are proving to be crucial for advancing our understanding of disease mechanisms and inter-tissue dynamics [249]. Bladder assembloids featuring an epithelial layer encasing a stromal core and an external muscular layer have been constructed to mimic the structural and functional properties of adult bladder tissue, including appropriate cell types, gene expression patterns, and regenerative potential [250]. To address the limitation of poor vascularization in organoids and assembloids, researchers have introduced endothelial and mesenchymal cells into brain organoid systems. Strategies include ETS variant transcription factor 2-driven endothelial differentiation and the incorporation of vascular organoids derived from hESCs [251,252,253].

Assembloids have also been employed to investigate inter-tissue communication. For instance, brain cancer modeling has been enhanced by combining region-specific human brain organoids with glioblastoma spheroids, improving the physiological relevance of preclinical models [254]. In another study, co-culturing anterior and posterior gut-derived organoids led to the emergence of hepato–biliary–pancreatic regions, highlighting the influence of spatial organization on organoid patterning [255]. Furthermore, the extent of fusion and ECM deposition has been shown to vary depending on the maturation stage of the organ-building block at the time of contact [256]. Collectively, these findings emphasize the potential of assembloids to replicate intricate multi-tissue interactions and disease-associated cellular dynamics.

#### 4.3.2. Bioprinting-Based Organoid Fabrication

Bioprinting technology offers a highly controlled and accurate platform for positioning and cultivating organoids, facilitating the fabrication of structured and reproducible 3D tissue models [257]. Recent progress in 3D bioprinting has shown the feasibility of arranging organoids into intricate architectures and fabricating larger-scale tissue constructs [258]. One notable study was conducted by Skylar-Scott et al., who developed an embedded bioprinting approach known as sacrificial writing into functional tissues (SWIFT) [259]. This method involves the direct printing of vascular networks into a dense, living 3D matrix composed of human iPSC-derived cardiac spheroids, enabling the formation of highly perfusable cardiac tissues.

Extruding bioinks that contain cell clusters or organoids has proven particularly effective. For example, vascular networks have been constructed by extruding cellular aggregates into strand-like forms [260]. In another approach, bovine colon organoids were embedded within gelatin methacryloyl (GelMA)-based bioink and printed onto 96-well plates to establish a high-throughput screening system that also supports cryopreservation [261]. Comparable strategies have utilized hydrogel-based bioinks containing spheroids to fabricate 3D cartilage-like tissue scaffolds, demonstrating the versatility of bioprinting in tissue engineering applications [262,263].

Advanced techniques, such as aspiration-assisted bioprinting and micromanipulation, have enabled more accurate handling of spheroids and organoids, allowing for their localization on matrix-coated surfaces or within self-healing hydrogel scaffolds [264,265]. These methods have proven useful for studying intercellular communication. For instance, Ayan et al. demonstrated that the precise arrangement of the spheroid—such as by positioning HUVEC spheroids near human dermal fibroblasts in fibrin—can trigger angiogenic sprouting [266]. Similarly, Daly et al. reconstructed post-myocardial infarction scarring by localizing cardiac fibroblast spheroids within engineered constructs [267].

Another innovative technique, known as spheroid transfer assisted by magnetic printing (STAMP), was developed [16]. This approach enables precise spatial control of delicate organoids by embedding magnetic nanoparticles within cellulose nanofiber hydrogels and utilizing a magnetized 3D printer, facilitating the assembly of neural and glioma organoids into three-dimensional brain cancer models. Similarly, another study introduced the Bio-Pick, Place, and Perfuse (Bio-P3) system, which utilizes large spheroids and honeycomb aggregates to create scalable tissue constructs [268]. The same research team further scaled this approach by assembling microtissues composed of up to 100 million HepG2 cells into larger tissue structures [269].

These precision-based “pick-and-place” strategies offer valuable tools for modeling complex tissue interactions and developing advanced drug screening platforms. However, their relatively slow fabrication speed remains a limitation for high-throughput or large-scale bioengineering applications.

One study demonstrated the fabrication of brain-like tissue by bioprinting neurospheroids within a bioink composed of alginate, gelatin, GelMA, and laminin into a GelMA-based support bath containing astrocytes and calcium [270]. To accelerate the fabrication of larger tissue constructs, another research team employed a volumetric bioprinting technique, which enabled the rapid production of intricate liver organoid-containing structures [271]. This approach involved projecting a 3D light pattern to simultaneously crosslink the entire organoid-laden GelMA construct on a rotating platform, eliminating the need for traditional layer-by-layer printing. Although still in early stages, 3D bioprinting of organoids presents considerable potential for constructing complex, multi-organoid tissue models.

Organoid-on-chip platforms have been further enhanced with features such as electrical stimulation, which promotes the functional maturation of cardiac spheroids and organoids, and integrated systems for the real-time monitoring of oxygen consumption to evaluate metabolic activity [4,272]. Overall, by customizing biochemical, mechanical, and electrical parameters to align with the unique requirements of each organoid type, these systems offer a more physiologically relevant environment, broadening their applicability for diverse biomedical investigations [273,274].

#### 4.3.3. Organ-on-Chip

Organoid-on-chip platforms integrate organoids with microfluidic technologies to replicate a dynamic and physiologically relevant in vitro environment. These systems emulate native tissue conditions by employing techniques, such as micropatterning of biochemical cues, mechanical stimulation, electrical inputs, soluble gradients, and controlled fluid flow, individually or in combination, to mimic organ-level functions [275]. For instance, introducing fluid shear stress within kidney organoid-on-chip models was shown to promote vascular network formation with permeable lumens and enhance tissue maturation, resulting in the development of proximal tubule and glomerular structures [276].

Fluid shear stress and perfusion have been shown to enhance the functional maturation of organoid-on-chip models across various tissues [277,278,279,280]. Introducing additional mechanical stimuli, such as cyclical pressure using a peristaltic pump in combination with perfusion, further replicates the physiological environment, particularly in the stomach. This dynamic simulation is especially beneficial for sustaining long-term organoid cultures by mimicking the mechanical forces naturally present in vivo [281].

## 5. Mechanistic Role of Hypoxia in Multilineage Communication and Disease Models

Hypoxia, a condition characterized by reduced oxygen availability, plays a pivotal role in embryonic development and organogenesis. In the context of organoid cultures, hypoxic conditions have been shown to affect multilineage communication, thereby affecting organoid maturation and functionality [41].

Generally, oxygen tension in most tissues ranges between 2% and 9%, with an average of approximately 3% [29]. As a result, numerous studies have focused on how hypoxia influences various aspects of stem cell biology, including their proliferation, differentiation capabilities, and gene expression patterns [282,283,284].

During embryogenesis, physiological hypoxia represents a critical regulator of cellular differentiation and tissue patterning. Similarly, in organoid cultures, hypoxia has been observed to modulate interactions among various cell lineages, including epithelial, mesenchymal, and endothelial cells. These interactions are essential for the development of complex tissue structures and the establishment of functional vasculature within organoids [285,286].

Recently, several studies have demonstrated the role of hypoxia in promoting vascularization within organoids. For instance, co-culturing mesodermal progenitor cells with neural spheroids under hypoxic conditions led to the formation of vascularized organoids, demonstrating the importance of oxygen gradients in facilitating endothelial and mesenchymal cell interactions [287].

Furthermore, hypoxia has been shown to influence the differentiation pathways of various cell types within organoids. A research team demonstrated how oxygen tension shapes the differentiation of human iPSC-derived liver buds (hiPSC-LBs), with a focus on its modulation of TGFB-mediated intercellular signaling during hepatogenesis [288]. This study highlighted the role of controlled hypoxia in influencing early liver development and enhancing organoid maturation in vitro. Mild hypoxia (10% O_2_) yielded the most significant and most numerous hiPSC-LBs, with enhanced albumin and vitronectin secretion, CYP3A4 activity, and urea production, indicating improved hepatoblast differentiation compared to excess hypoxia or ambient conditions [288]. This oxygen level also preserved stromal cells (HUVECs and MSCs) and suppressed TGFB1, TGFB3, and INHBA expression, fostering a pro-hepatocytic environment. TGFB1 and TGFB3 were predominantly produced by mesenchymal and endothelial cells in both fetal liver and hiPSC-LBs, identifying stromal cells as a primary TGFB source. Excess hypoxia upregulated TGFB family genes and cholangiocyte markers while reducing albumin secretion [288]. In sum, this report concluded that controlled oxygen delivery, particularly mild hypoxia, promotes stable hepatocyte differentiation in hiPSC-derived liver buds by modulating TGFB signaling from mesenchymal and endothelial cells. Both excessive TGFB activity under strong hypoxia and excessive suppression hinder hepatocyte maturation. Replicating physiological oxygen conditions alongside multicellular co-culture may enhance liver organoid maturation in vitro, supporting applications in regenerative medicine and drug screening.

A study examined hiPSC-LBs to compare 3D multilineage organoid interactions with conventional 2D differentiation [42]. In 2D, iPSCs were sequentially differentiated into hepatocyte-like cells (day 0–21), serving as a monoculture baseline. In contrast, 3D LBs were generated by co-culturing iPSC-derived hepatic endoderm with mesenchymal (MC) and endothelial cells (ED), which self-organized into tissue within 24 h.

Hepatic cells in LBs followed distinct lineage trajectories, showing intermediate maturation with epithelial migration signatures (e.g., PROX1, ONECUT2) similar to fetal hepatoblasts [42]. Transcriptomic analyses revealed that LB cells, ECs, and MCs closely resembled fetal liver lineages and engaged in extensive crosstalk, with MCs showing the strongest interactions. Pathway analyses identified TNF, FGF, JAK/STAT, NF-κB, HIF, and VEGF as key drivers; notably, VEGFR2 inhibition impaired endothelial sprouting and hepatoblast differentiation in 3D but not 2D systems.

LBs displayed hypoxic stress (HIF-1α upregulation) in vitro, which was rapidly resolved after transplantation into mice, as vascularization was established within 48 h, promoting hepatic maturation. Hypoxia activates multiple signaling pathways (TNF, FGF, JAK/STAT, NF-κB, HIF, VEGF) and induces extracellular matrix production along with remodeling enzymes (MMP2/4/19) [42]. Together, these changes shape the organoid microenvironment, promoting multilineage communication that is critical for hepatocyte maturation.

The interplay between hypoxia and multilineage communication is also evident in disease modeling. In cerebral organoids, chronic hypoxia was found to remodel the TME, supporting the expansion of glioma stem cells and highlighting the role of oxygen availability in tumor progression [289].

These findings underscore the significance of hypoxia as a modulator of multilineage interactions in organoid systems. By carefully controlling oxygen levels, researchers can enhance the physiological relevance of organoid models, thereby improving their utility in studying human development and disease.

Hypoxia is a crucial factor that impacts the survival and successful engraftment of islets following islet transplantation [290]. Islets, especially β-cells, have limited capacity to cope with low oxygen levels and oxidative stress due to their naturally low antioxidant enzyme expression [291]. Although the body initiates natural responses to promote neovascularization, the formation of new blood vessels typically takes around 7 to 14 days, during which a significant proportion of islets, particularly the larger ones, fail to survive [292]. The low oxygen availability at common implantation sites, such as the liver, further exacerbates this challenge. Encouragingly, several strategies aimed at promoting vascularization and enhancing islet viability, even within challenging sites, such as the subcutaneous space, have yielded promising results, demonstrating strong potential for clinical translation [293,294].

Liu et al. developed a bioelectronic heart-on-a-chip platform to study the impact of acute hypoxia on cardiac function [4]. This system integrates microfluidics with extracellular or intracellular bioelectronics, enabling rapid oxygen modulation to simulate coronary occlusion and reperfusion by switching between normoxic (21% O_2_), hypoxic (1–4% O_2_), and recovery (21% O_2_) conditions. Using HL-1 mouse atrial cells, the model effectively reproduced normoxia, hypoxia, and early recovery phases. Hypoxia (1% O_2_ for 2.5 h) induced strong nuclear HIF-1α expression, which normalized after 90 min of reoxygenation. Acute hypoxia (1% O_2_ for 5 h) initially caused tachycardia (beating rate from 3.2 to 4.2 Hz), followed by arrhythmia with prolonged firing intervals (4.9 ± 1.5 s), mirroring responses in primary cardiomyocytes. This platform offers real-time, multiplexed readouts, making it valuable for studying hypoxia, ischemia, and reperfusion injuries, as well as for drug testing [4]. The authors propose future enhancements, including the incorporation of ischemia-related factors (e.g., acidosis, hyperkalemia), expanded multiplexing, stable long-term intracellular recordings, and adaptation for 3D cardiac tissues.

Hypoxia serves as a major driver of numerous cancer-related processes, including enhanced aerobic glucose metabolism [295,296], activation of angiogenesis via factors like VEGF and vascular endothelial growth factor receptor (VEGFR) [297,298], the initiation of EMT pathways [299,300,301], and the engagement of immune cells that support tumor growth such as myeloid cells [302], regulatory T cells [303], and tumor-associated macrophages [304]. Although hypoxia plays a crucial role in tumor progression, conventional 2D cell cultures fail to replicate the hypoxic conditions of the TME accurately [305]. In contrast, 3D models, including spheroids, organoids, 3D scaffolds, and microfluidic systems, have been shown to better mimic this key characteristic of the TME in numerous studies [306,307,308].

Zhi et al. developed a cortical organoid-on-a-chip model that mimics the physiological hypoxia of the human fetal brain to study neural differentiation and assess the effects of Tanshinone IIA (Tan IIA) [220]. The platform integrates a microfluidic chip with a micropillar array, enabling controlled formation of cortical organoids from hiPSCs. Featuring four independent chambers, the chip supports efficient drug screening. Embryoid bodies seeded onto the chips differentiated into millimeter-sized cortical organoids under specific culture conditions.

To simulate in vivo hypoxia, organoids were cultured in 5% O_2_ for 30 or 50 days. The model successfully replicates the hypoxic microenvironment of early brain development, enhancing neurogenesis, synaptogenesis, and neuronal maturation compared to normoxic conditions [220]. Tanshinone IIA (Tan IIA), a traditional Chinese herbal medicine, further promoted neural progenitor differentiation, neuronal activity, and functional network formation. This organoid-on-a-chip system offers a powerful tool for studying human cortical development under hypoxia and for evaluating neurotherapeutics.

Hypoxia in the core regions of larger organoids, caused by the lack of functional vasculature, often leads to nutrient deprivation, necrosis, and diminished functionality [309]. To fully realize the therapeutic potential of these 3D organoid systems, it is essential to engineer sophisticated vascular networks within their structure [310].

In organoids, the development and branching of blood vessels are finely orchestrated by spatial gradients of pro-angiogenic factors, a complexity that is challenging to replicate within organoid systems [310]. Hypoxic conditions stimulate the formation of a VEGF gradient, where cells in low-oxygen regions produce higher levels of VEGF compared to well-oxygenated areas. Endothelial cells can sense these VEGF signals through VEGFR2 and initiate a Notch-dependent lateral inhibition mechanism. This signaling cascade designates specific cells with elevated VEGFR2 expression as tip cells to guide the sprouting process along the VEGF gradient, while adjacent stalk cells, characterized by increased VEGFR1 expression, contribute to vessel elongation [310].

A recent study developed the vascular network quality index (VNQI), a standardized and quantitative metric to evaluate the physiological relevance of vascularized microphysiological systems (vMPS) based on oxygen transport [311]. This study addresses the limitations of current user-dependent, non-standardized, and morphology-based assessments that poorly reflect oxygenation function. The researchers generated a dataset of 500 diverse vMPS samples, varying parameters such as endothelial cell source and density, fibroblast density, growth factor levels, and ECM stiffness [311]. Vascularized islet-chips with higher VNQI scores preserved and enhanced insulin secretion under hypoxic conditions, unlike poorly vascularized controls.

A chained neural network effectively derived the VNQI, which correlated strongly with actual oxygen transport. This metric offers a robust, standardized tool for evaluating vascularization in vMPS, bridging morphology and function through the application of machine learning.

Low-oxygen conditions have been demonstrated to contribute to the development of fetal organs and the placenta. However, it has also been found that hypoxic culture conditions, by themselves, are insufficient to promote progenitor cell expansion [288]. A recent study tackled the challenge of limited size and functionality in hiPSC-derived liver organoids, which has restricted their potential in regenerative medicine [312]. In this study, researchers explored progenitor expansion during mouse liver development to identify key factors that could enhance hiPSC-liver organoid growth. They focused on the interaction between the placenta and fetal liver, visualizing placenta-derived blood perfusion by injecting AlexaFluor647-conjugated lectin and anti-CD31 antibody through the umbilical vein. Using confocal microscopy, the researchers generated 3D images and quantified liver vasculature at embryonic stages E9.5, E10.5, and E11.5.

The researchers found that placenta-derived perfusion began at E10.5, primarily targeting the ventral lobes, which subsequently grew 9.1-fold by E11.5 compared to 3.7-fold for dorsal lobes [312]. Notably, despite active perfusion, the liver remained hypoxic at E10.5 and became oxygenated only by E11.5, when hepatoblasts transitioned to oxidative metabolism, highlighting the role of oxygen in growth post E11.5. In line with these findings, IL1α treatment under hypoxia significantly promoted the expansion of hiPSC-liver organoids and hepatoblasts (ALB^+^CK19^+^). The study effectively recreated the transient placenta–liver interaction by sequentially supplying placenta-derived IL1α under hypoxia, followed by oxygenation [312]. This approach, which mimics the physiological oxygen profile and IL-1α signaling, was identified as critical for optimal progenitor expansion and organoid development.

Kakni et al. developed an innovative in vitro model, hypoxia-tolerant apical-out human small intestinal organoids, to study host–microbiome interactions under physiologically relevant conditions [313]. This model addresses the limitations of existing gastrointestinal systems that fail to replicate the low-oxygen environment essential for the dominant anaerobic gut microbiota. Human intestinal organoids were efficiently differentiated under hypoxia (5% O_2_), maintaining expected cellular composition and gene expression profiles for proliferation, differentiation, and mesenchymal markers, comparable to normoxic and Matrigel-embedded organoids. The organoids actively responded to hypoxia, with elevated HIF-1α protein expression and significant upregulation of HIF-1α target genes linked to mucosal barrier function (e.g., MUC2, MUC3A, TFF3, KRT20, SLC2A1). The hypoxic apical-out organoids exhibited intact epithelial barrier integrity and polarized nutrient absorption, effectively excluding 4 kDa FITC-dextran and absorbing fluorescent fatty acids from the medium [313]. This study presents the first human small intestinal organoid model with reversed polarity, capable of sustained culture and function under hypoxic conditions (5% O_2_).

Kip et al. developed and characterized human small intestinal organoids (hSIOs) as a multicellular 3D model to investigate intestinal ischemia–reperfusion (IR) injury [314]. Unlike conventional 2D cell lines, hSIOs more accurately mimic the cellular complexity and architecture of the intestinal epithelium. The study differentiated hSIOs into crypt-like (CL) organoids with proliferative features and villus-like (VL) organoids enriched in differentiated enterocytes and goblet cells, emulating the distinct susceptibilities of crypts and villi to IR injury in vivo [314]. VL organoids showed pronounced cellular stress responses, with increased expression of oxidative stress markers (e.g., CHCHD2, ERO1A), pro-apoptotic proteins (e.g., DIABLO, LCN2, BCAP31), and complex I subunits linked to elevated ROS production. Enriched processes were selectively enriched in VL organoids, including the upregulation of basement membrane components, which is consistent with the early damage observed at villus tips during IR injury. Kim et al. developed 3D neural organoids derived from adult fibroblast–reprogrammed neural stem cells, which structurally mimic the human cerebral cortex and enable the modeling of hypoxic brain injury [315]. Following induced hypoxia and reoxygenation, the organoids showed restored neuronal proliferation but impaired maturation, highlighting their potential for neurodegenerative disease research and tailored drug evaluation.

Physiological hypoxia has been shown to enhance the growth and functional maturation of human intestinal epithelial organoids. Under oxygen levels of 2% compared to 20%, these cultures exhibited altered gene expression profiles associated with differentiation, metabolism, mucus barrier formation, and immune regulation [34]. Oxygen concentration significantly influences hPSC hepatic differentiation outcomes and should be regarded as an essential translational variable in scalable liver organoid systems for therapeutic and pharmacological use. Table 2 summarizes representative studies utilizing hypoxia-modulated organoid-on-chip platforms across various organ systems. These examples demonstrate how controlled low-oxygen environments have been integrated into advanced 3D microphysiological systems, including bioelectronic, microfluidic, and vascularized chips, to mimic in vivo physiology and investigate organ-specific responses. By applying defined hypoxic conditions, these models have provided new insights into cardiac function, neurodevelopment, and endocrine regulation, underscoring the importance of oxygen tension in shaping tissue maturation, function, and disease phenotypes.

**Table 2:** Hypoxia-associated disease models in organoids.

Organoid Model	3D System Used	Hypoxic Condition	Key Findings	Ref.
Cardiac Organoids	Bioelectronic chip	Acute hypoxia (1% O_2_ for 2.5–5 h)	Hypoxia led to tachycardia and arrhythmia, mimicking real-time responses in primary cardiomyocytes.	[4]
Cortical Organoids	Microfluidic chip	5% O_2_ for 30 or 50 days	Physiological hypoxia can simulate a prenatal environment, explore brain development, and screen natural neuroactive components. Hypoxia enhances neurogenesis, synaptogenesis, and neuronal maturation.	[220]
Vascularized islet-on-chip model,	Developed a vascularized microphysiological system (vMPS) and introduced the vascular network quality index (VNQI)	−96 h culture → image islets, refresh media → 5% O_2_ for 4 h → add 10 mM glucose media → 1 h hypoxia	Higher VNQI scores were preserved. Enhanced insulin secretion under hypoxia, demonstrating a link between vascular quality and function.	[311]
Human intestinal organoids	Small intestinal organoids from hindgut spheroids. Unraveling mechanisms related to host–microbiome interactions and developing microbiome-related probiotics and therapeutics.	5% O_2_	Hypoxia-tolerant “apical-out” organoids maintained their cellular composition and barrier function, with elevated HIF-1α expression and upregulation of barrier-related genes. HIF-1α-related mechanism	[313]
Liver organoids	hPSC-derived liver bud model	Utilizing an O_2_-permeable polydimethylsiloxane (PDMS) culture plate: Mild hypoxia (10% O_2_, permeable plate): Minimum dissolved O_2_: ~13% Ambient (20% O_2_, permeable plate): Minimum dissolved O_2_: ~19% Excess hypoxia (20% O_2_, non-permeable plate): Lowest dissolved O_2_: ~10% for first 3–4 days, gradually rising afterward	Hypoxic conditions enhanced the maturation of hepatocyte-like cells. Promoting the development of the bile duct. Regulating TGFBR ligand expression. Controlled hypoxia-driven TGFB signaling supports optimal hepatoblast maturation.	[288]
Intricate human organoid models	Complex human organoid models incorporating vascular networks were generated by integrating hPSC-derived mesodermal progenitor cells (MPCs).	20% O_2_ and 2% O_2_	Under normoxia (20% O_2_), vascular networks clustered asymmetrically within spheroids. Under hypoxia (2% O_2_), uniform capillary-like endothelial growth was promoted throughout the organoid, likely driven by pro-angiogenic factors such as VEGF and HIF1α activation.	[287]
Cerebral organoids	Long-term glioma cerebral organoids (ltGLICOs) for studying glioblastoma within a human brain tumor microenvironment.	Standard organoids (lacking patient-derived glioma stem cells) were cultured at 5% O_2_ to assess how hypoxia influences cellular composition and pro-tumorigenic ligand release.	Chronic hypoxia remodels the TME, supporting the expansion of glioma stem cells. Age-related cerebral vascular decline and chronic regional hypoxia are thought to drive the aggressive expansion of glioma stem cells in glioblastoma.	[289]
Human small intestinal organoids	hSIOs were cultured as: Crypt-like (CL): Grown in growth medium (GM) for 12 days, yielding a proliferative, stem cell-rich phenotype. Villus-like (VL): Cultured in GM for 7 days, then in differentiation medium (DM) for 5 days, producing enterocyte- and goblet–cell–enriched organoids.	Intestinal ischemiareperfusion injury was modeled by exposing organoids to 12 h hypoxia (<1% O_2_, 5% CO_2_), followed by 30 min or 120 min of reoxygenation (21% O_2_, 5% CO_2_).	Crypt-like and villus-like structures showed distinct susceptibility to injury. Villus-like organoids exhibited pronounced cellular stress, oxidative stress, and pro-apoptotic responses, mimicking in vivo damage. Different hypoxia–reoxygenation responses in crypt-like versus villus-like intestinal epithelial cells	[314]

**Figure 6:**
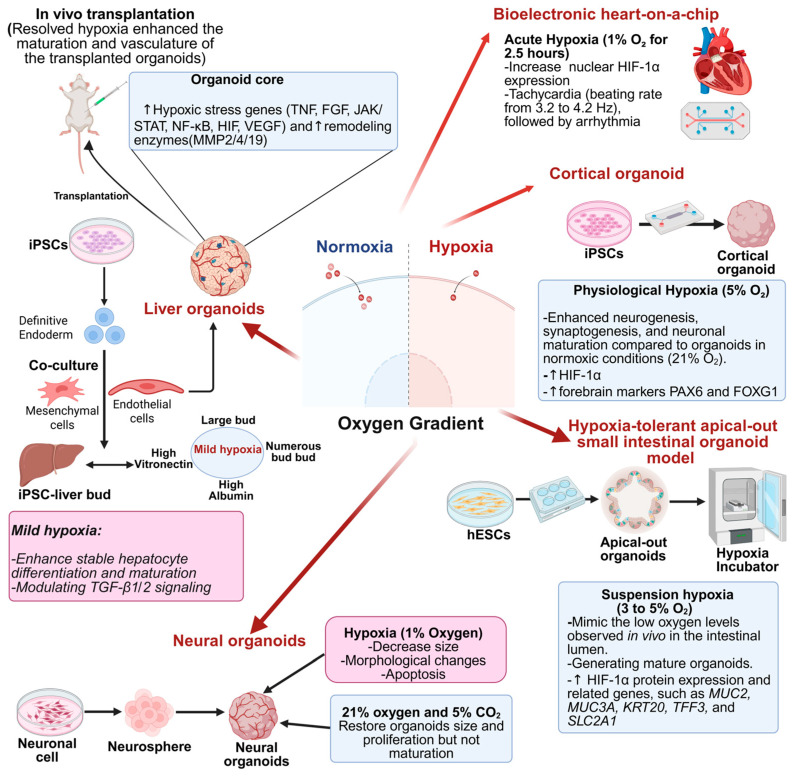
Schematic diagram depicting how hypoxia shapes the development, maturation, and functional responses of organoids across multiple systems. Created with BioRender.com. Abbreviation: ↑: upregulation.

Taken together, controlled hypoxia emerges as a double-edged regulator in organoid systems. On the one hand, insufficient oxygen can induce cellular stress, apoptosis, and impaired maturation, limiting organoid viability. On the other hand, carefully tuned physiological hypoxia enhances multilineage communication, stimulates vascularization, and drives tissue-specific differentiation, thereby increasing the physiological relevance of organoid models. By harnessing hypoxia-responsive signaling pathways and integrating oxygen-engineering strategies, researchers can overcome inherent limitations and generate advanced platforms for studying development, disease mechanisms, and therapeutic applications. Figure 6 illustrates this concept, highlighting both the detrimental consequences of excessive hypoxia and the beneficial outcomes of controlled oxygen regulation, underscoring its pivotal role in engineering functional and clinically relevant organoids.

## 6. Engineering 3D Models Under Hypoxia: Tools and Approaches

Current research is now being directed towards the use of 3D models as an application for in vitro modeling, to enhance cellular interaction, aiming for interactions similar to those observed in vivo models [316,317]. The 3D models include spheroids, organoids, or hydrogels [318]. To obtain an optimum 3D model, optimization of both the 3D matrix and the oxygen concentration is crucial [318]. Although well established in 2D models, PO_2_ in 3D systems remains not fully understood, due to the abnormalities and variety in cellular structure thickness that impede gas passage and generate oxygen concentration gradient, as reported [319].

Egger et al. presented a high-throughput cell-encapsulated hydrogel system made of polyether ketone that helped to establish a hypoxic/anoxic environment [318]. Standard hydrogel crosslinking methods, such as GelMA, are known to consume oxygen during the process [319,320]. This challenge can be addressed by using oxygen-permeable biomaterials, which enable the controlled and sustained release of oxygen to enhance tissue repair efficacy [321]. Oxygen release can be achieved either through oxygen generation, such as by employing photosynthetic algae [321,322,323], or by using peroxides [321,324]. To ensure sustained oxygen delivery and prevent cytotoxicity from rapid peroxide decomposition, the encapsulation of peroxide is required [324]. Among these peroxides is the micro-sized calcium peroxide, which was loaded on polylactic acid and embedded into the silk fibroin and oxidized pectin hydrogel, where its effect lasted for 14 days in bone models [325].

Another study by Tehrani et al. fabricated a chitosan/β-glycerol phosphate thermosensitive hydrogel enclosing polylactic acid as a capsule to enclose hydrogen peroxide, along with polyvinylpyrrolidone; it was coated with a layer of decellularized human amniotic membrane for wound healing that sustained oxygen release for a week [326]. Nevertheless, the use of peroxides still has the drawbacks of the optimizations required to maintain oxygen release, as well as bio-incompatibility and toxicity that can result from the fabricated hydrogels [321]. In addition to oxygen generation, oxygen release can be accomplished via the oxygen-carrying system that involves the loading of oxygen on hydrogels and their release to the desired site by the integration of hemoglobin [327,328] or perfluorocarbons [329,330,331] into the hydrogels.

The 3D organoid model provides a physiologically relevant platform for replicating HIF-1α–regulated metabolic processes in hepatocellular carcinoma (HCC). Unlike chemical induction with cobalt chloride (CoCl_2_), this model naturally recreated hypoxic conditions and the associated shift to HIF-1α–dependent glycolysis [332].

During organoid development, hypoxia favored the selection of oxygen-tolerant cell populations, which may underline the malignant characteristics of pancreatic ductal adenocarcinoma (PDAC) [333]. This phenomenon provides a valuable platform for investigating disease mechanisms and identifying novel therapeutic strategies. Physiological hypoxia enhanced both the growth and functional maturation of human intestinal epithelial organoids. Specifically, lowering oxygen levels from 20% to 2% in colon-derived differentiated intestinal organoids altered gene expression profiles associated with differentiation, metabolism, mucus barrier formation, and immune regulation, thereby providing a valuable platform for disease modeling and personalized drug testing [34].

Colonoids cultured at 2% oxygen developed greater cell mass than those at 20%. Growth was similar until day six, after which differentiated colonoids showed enhanced expansion at 2% oxygen. In both oxygen conditions, colonoids proliferated and differentiated into goblet, absorptive, and enteroendocrine cells [34]. Undifferentiated colonoids expressed higher levels of the proliferation marker Ki67, while differentiated colonoids had elevated MUC2 (goblet) and CK20 (absorptive) expressions, with a trend toward more CGA-positive enteroendocrine cells. Protein-level expressions of Ki67, MUC2, CK20, and CGA did not differ significantly between oxygen levels, indicating that oxygen tension does not substantially alter intestinal epithelial cell composition [34]. Collectively, physioxia (2% oxygen) is preferable for colonoid research when closely replicating in vivo conditions. Then, human colonoids can be cultured from single cells in continuous low oxygen, often performing better than in 20% oxygen.

A recent study examined how oxygen concentration during the initial establishment of 3D pancreatic cancer organoids (PCOs) influences their properties, aiming to minimize selection bias from conventional normoxic culture and more accurately model the hypoxic tumor microenvironment in vivo [333]. The researchers utilized human pancreatic ductal adenocarcinoma (PDAC) surgical specimens to generate 3D organoids. The same tissue samples were digested into single cells and cultured from the outset under normoxia (20% O_2_, producing NORMO-PCOs) or hypoxia (1% O_2_, producing HYPO-PCOs) [333]. To evaluate phenotype stability, NORMO-PCOs were later switched to 1% O_2_ and HYPO-PCOs to 20% O_2_ for three passages before further analysis.

Both normoxic and hypoxic pancreatic cancer organoids were successfully established and maintained for over 20 passages. NORMO-PCOs displayed glandular morphology, while HYPO-PCOs formed solid structures. The source PDAC tissue contained both moderately and poorly differentiated regions; NORMO-PCOs reflected the well-differentiated areas with strong E-cadherin, absent vimentin, and high GATA6, whereas HYPO-PCOs mirrored the poorly differentiated regions with weak E-cadherin, high vimentin, and minimal GATA6 [333]. These phenotypic patterns were reproduced in xenografts, where NORMO-PCOs formed adenoductal structures and HYPO-PCOs produced undifferentiated regions, indicating oxygen-driven cell type selection. Transcriptomic profiling revealed distinct signatures: NORMO-PCOs were enriched for Moffitt classical signaling, and HYPO-PCOs for epithelial–mesenchymal transition (EMT) and Moffitt basal-like signaling [333]. HYPO-PCOs expressed higher VIM, S100A2, and TP63, and lower CDH1 and GATA6, with PurIST scoring confirming classical and basal-like subtype classifications for NORMO- and HYPO-PCOs, respectively.

An organoid system generated from human glioblastomas successfully replicated the hypoxic gradients and cancer stem cell heterogeneity seen in vivo, providing a platform to study both microenvironmental dynamics and cancer stem cell (CSC) biology simultaneously [306]. Physiological hypoxia enhanced vascular development in iPSC-derived kidney organoids. Culturing under 7% O_2_ increased average vessel length, which correlated with upregulation of VEGFA-189 and VEGFA-121 and suppression of the anti-angiogenic isoform VEGF-A165b [334].

In prevascularized human cardiac organoids, stabilization of HIF-α represents an effective strategy to enhance endothelial cell expression and lumen development, providing a strong foundation for advancing prevascularization approaches in cardiac and other tissue transplantation therapies [335]. Furthermore, HIF-1α activation promoted vascular network formation in kidney organoids and conferred protection against cisplatin-induced injury under hypoxic conditions [336].

In iPSC-derived kidney organoids, hypoxia promoted vascularization by extending vessel length, elevating VEGFA-189 and VEGFA-121, and downregulating VEGF-A165b. This underscored the role of hypoxic culture in improving organoid vascular networks [334]. Moreover, lung tumor organoids exhibited hypoxic conditions that modulated the expression of lung cancer markers, including thyroid transcription factor-1, cytokeratin 7, and the ΔNP63 variant, as well as the upregulation of stem cell-associated markers, resulting in an increased proportion of cancer stem cells. Furthermore, these exhibited unique migratory behavior upon reoxygenation, driven by epithelial-mesenchymal transition and the elevated expression of matrix metalloproteinases 7 and matrix metalloproteinases 9, indicating their enhanced invasive potential [337]. However, in breast cancer 3D models, both hypoxic and normoxic conditions produced cellular phenotypes closely resembling those grown on 3D-printed scaffolds, whereas cells cultured in 2D or within Matrigel displayed distinct characteristics independent of oxygen levels [338].

**Figure 7:**
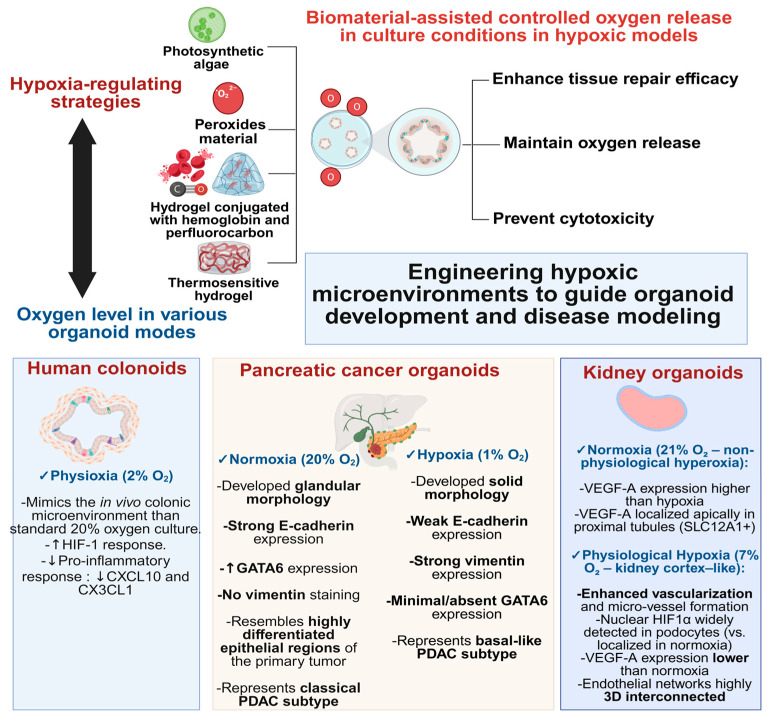
Engineering hypoxic microenvironments to guide organoid development and disease modeling. A schematic illustrating a strategy to regulate hypoxia in vitro using biomaterials (photosynthetic algae, peroxides, perfluorocarbons, and thermosensitive hydrogels) that enable controlled oxygen release, enhance tissue repair, and minimize cytotoxicity. The lower panels demonstrate the impact of oxygen tension on different organoid systems: (i) human colonoids cultured under physioxia (2% O_2_) replicate in vivo colonic microenvironments and reduce inflammatory responses; (ii) pancreatic cancer organoids exhibit distinct morphologies under normoxia (glandular, classical PDAC subtype) versus hypoxia (solid, basal-like PDAC subtype), recapitulating tumor heterogeneity; and (iii) kidney organoids display improved vascularization and 3D endothelial networks under physiological hypoxia (7% O_2_) compared to normoxia (21% O_2_). These models underscore the role of hypoxia engineering in enhancing organoid maturation, functionality, and disease relevance. Created with BioRender.com. Abbreviasions: ↑: upregulation; ↓: down regulation; ✓: Condition occurring.

In summary, controlled hypoxia is a pivotal factor in replicating in vivo physiology within organoid systems. Advances in biomaterial-based oxygen delivery now enable precise regulation of oxygen levels, ensuring a balance between cell survival, maturation, and functional performance. As summarized in Figure 7, the engineering of hypoxic microenvironments provides a powerful strategy to bridge the gap between in vitro and in vivo systems, laying the foundation for more accurate models in regenerative medicine, disease research, and therapeutic discovery.

## 7. Optimizing Organoid Systems: Hypoxia-Mediated Multilineage Interaction

Multilineage organoids are offering a platform that helps to mimic organogenesis during embryonic development, opening doors for their use as disease models in studies [339]. Among the most studied multilineage organoids were liver organoids [340]. A study performed by Takebe et al. developed a robust protocol for generating interconnected hepatic, biliary, and pancreatic (HBP) structures from hPSCs by fusing anterior and posterior gut spheroids [341]. The resulting multi-organ model mimics in vivo organogenesis and offers a promising platform for studying development, disease, and regenerative therapies. Guan et al. engineered an autosomal recessive polycystic kidney disease (ARPKD) mutated multilineage liver organoid to study hepatic fibrosis, showing elevation in collagen production and offering a fibrotic pathology, offering a model that can be used for further fibrosis drug screening [232]. Multilineage live organoids have attracted increasing attention from researchers as powerful models for studying disease. Goulart et al. proved that multilineage liver organoids produce more albumin and metabolic enzymes, offering another model with which to study different pathways of liver development [342].

Camp et al. advanced multilineage differentiation by employing single-cell RNA sequencing before organoid formation, demonstrating that the resulting model exhibits a gene expression profile comparable to that of the developing fetal liver [42]. With a special interest in studying fetal organ development, Branco et al. created a multilineage pro-epicardium, which gives rise to the outermost layer of the heart [343]. In this study, the pro-epicardium organoids were co-cultured with cardiomyocytes, giving rise to a functional heart organoid to help further study the crosstalk between myocardium and epicardium in health and disease [343].

Voges et al. demonstrated that human cardiac organoids (hCOs), which mimic immature heart tissue, can fully recover function after injury without fibrosis or hypertrophy, indicating a regenerative capacity similar to that seen in neonatal hearts [344]. Using a cryoinjury model, they showed that the injured cardiac tissue could achieve complete functional recovery within 14 days, highlighting the intrinsic regenerative potential of the immature heart.

Researchers developed heart-forming organoids (HFOs) from human pluripotent stem cells using Matrigel embedding and WNT pathway modulation, resulting in complex structures that mimic early heart development [345]. These organoids not only model key cardiac architecture and tissue interactions but also enable the in vitro analysis of genetic defects, as shown by NKX2.5 knockout-induced malformations. Another studied organ was the lung, where Miller et al. recruited hiPSCs to form both human lung organoids resembling the bronchi and bud tip progenitor organoids [346]. Both types of respiratory tract organoids proved to be representative of human fetal-like tissue, offering a model with which to investigate cellular crosstalk and epithelial fate throughout lung development [346]. de Carvalho et al. presented a 3D collagen I culture system that enables multilineage maturation of hPSC-derived lung progenitors into both proximal and distal airway cells by modulating GSK3, NOTCH, and WNT signaling [347,348]. The findings reveal key mechanisms that drive lung cell differentiation and provide a platform for advancing human lung development models.

Peng et al. reported that the overexpression or stabilization of HIF-1α enhances vascularization in kidney organoids derived from human iPSCs and enhances protection against cisplatin-induced injury under hypoxic conditions [336]. By improving endothelial cell development and activating antioxidant pathways, HIF-1α supports both organoid maturation and their potential for disease modeling. These findings highlight HIF-1α as a promising target for enhancing the physiological relevance and therapeutic utility of kidney organoids.

Using hypoxia-induced differentiation, Lim et al. established kidney organoids with interconnected collecting ducts and nephrons, enhancing structural and functional maturity [349]. One of the diseases where hypoxia plays a primary role is cancer. Establishing hypoxic organoids enhances the replication of TME. Ziółkowska-Suchanek reviewed the influence of hypoxia on lung organoids, highlighting current model limitations, particularly inadequate surface diffusion [350]. Dauer et al. also reviewed the impact of hypoxia on cancer and concluded that, in pancreatic adenocarcinoma, hypoxia is more closely associated with chemoresistance than with proliferation or angiogenesis, underscoring its relevance in chemotherapy drug screening [351].

A research report introduced a scaffold-free 3D cartilage regeneration approach using cartilaginous organoid bio-assembly (COBA) [352]. The method consistently achieved efficient production, structural integration, and functional restoration. Mechanistic analysis revealed that cell–cell adhesion proteins drive aggregation and cytoskeletal reorganization during organoid formation, while hypoxia within dense aggregates activates HIF-1α–mediated glycolysis to sustain the chondrogenic phenotype and enhance extracellular matrix deposition. In vivo, separated cartilaginous organoids fused into continuous cartilage, enabling applications, such as minimally invasive nasal dorsum augmentation, in goats [352]. Although a proof of concept, these findings highlight COBA’s potential for clinical translation in plastic and reconstructive surgery.

This study introduces a 3D multicellular neurovascular unit (NVU) organoid that models hypoxia- and inflammation-induced blood–brain barrier (BBB) dysfunction, providing a physiologically relevant platform for neurological disease research, including ischemic stroke [353]. The approach addresses the limitations of conventional and animal models, which often fail to replicate the human BBB structural and functional complexity. In this study, the researchers generated a human cell–based 3D neurovascular organoid model composed of six cell types (brain microvascular endothelial cells, pericytes, astrocytes, microglia, oligodendrocytes, and neurons), using a staged assembly process with hanging-drop culture. The cell ratios were precisely controlled (e.g., 30% endothelial cells, 15% pericytes, 15% astrocytes) [353].

To mimic ischemic stroke, organoids were exposed to 0.1% O_2_ for 24 h in a hypoxic chamber and compared with normoxic controls. BBB function was assessed by tracking FITC-labeled IgG, albumin, and dextran permeability, while cell viability was measured via calcein AM and ethidium homodimer-1 staining. Hypoxic organoids demonstrated greater permeability to large molecules (IgG, albumin) than normoxic controls, indicating BBB compromise [353]. These results concluded that the multicellular 3D neurovascular unit organoid reliably models BBB dysfunction under hypoxic and neuroinflammatory conditions, offering a robust in vitro platform for disease research and therapeutic testing.

## 8. Conclusions and Future Perspective

Organoid research offers a more physiologically relevant in vitro representation of human biology, with features such as hypoxia and multilineage differentiation, which are shown to influence cellular behavior and immune responses. These attributes enhance the fidelity of disease models and their utility in drug screening. However, challenges remain, particularly in scalability and vascularization.

Although organoids hold great promise for therapeutic and clinical applications, several limitations currently restrict their broader utility. Their small size is advantageous for high-resolution imaging and AI-driven analysis. However, it hinders translation into transplantable or clinically relevant constructs. Organoids, particularly those modeling complex tissues, such as the brain, often exhibit heterogeneity and batch-to-batch variability in size, architecture, and maturation, which impairs reproducibility and large-scale automation. Dependence on manual handling and biological matrices, such as Matrigel, further complicates workflow standardization. Moreover, high-content imaging generates computationally intensive datasets; some organoid types are slow-growing or difficult to establish, limiting throughput. Consequently, issues related to scalability, vascularization, and standardization remain significant barriers to the widespread incorporation of organoids into high-throughput biomedical platforms.

Hypoxia-modulated organoids development affords a robust translational potential in personalized medicine, drug development, and regenerative therapy. By reconstructing tissue-specific microenvironments, including physiologically relevant low-oxygen conditions, they offer more accurate platforms for predicting therapeutic responses. In precision oncology, patient-derived tumor organoids cultured under hypoxic conditions enable the pre-clinical testing of individualized drug regimens to optimize efficacy and minimize toxicity.

For drug discovery, incorporating hypoxic conditions improves the identification of compounds effective against resistant cell populations and better emulates clinical metabolic and molecular tumor profiles. In regenerative medicine, low oxygen-conditioned organoids enhance stemness, differentiation, and repair capacities, enabling the modeling of hypoxia-induced injury responses (e.g., brain and intestinal damage) and the development of tissue-specific therapeutic strategies. Overall, oxygen modulation improves the physiological relevance of organoid systems, enhancing their value for clinical translation.

This review highlights two critical microenvironmental factors, hypoxia and multilineage communication, which impact organoid behavior and their utility in disease modeling. Hypoxia, primarily mediated by HIFs, is not merely a passive condition of 3D culture but a potent regulator of cell fate, metabolism, and gene expression. Deliberate modulation of hypoxic conditions enables more accurate modeling of pathological states such as ischemia, cancer, and fibrotic diseases. Concurrently, multilineage communication is driven by complex intercellular signaling; ECM remodeling enhances the structural and functional complexity of organoids, allowing for a more faithful recapitulation of in vivo multicellular interactions, including immune dynamics and tissue regeneration.

Recent advancements in the engineering of hypoxic niches and co-culture systems have substantially improved the physiological and immunological relevance of organoid models. These innovations have broadened the translational potential of organoids in applications such as high-throughput drug screening, regenerative medicine, and precision therapeutics. Nevertheless, key challenges persist, including the need for standardized protocols, improved vascularization, and the incorporation of systemic physiological cues to fully replicate in vivo conditions.

Incorporating the dynamic, spatially controlled hypoxic gradients alongside programmable ECMs will allow for more precise modeling of tissue- and disease-specific microenvironments. In parallel, the integration of multi-omics technologies with computational tools, such as single-cell multi-omics, spatial transcriptomics, and machine learning, is poised to deepen our understanding of lineage commitment, intercellular communication, and the molecular mechanisms underlying disease. Additionally, the development of perfusable, vascularized organoids that integrate immune components will significantly enhance their physiological relevance, particularly for modeling systemic diseases and investigating immuno–oncology interactions. In the realm of precision medicine, patient-derived organoids combined with CRISPR-based gene editing and high-throughput drug screening hold great promise for advancing personalized therapies and predictive diagnostics.

Integrating patient-derived cells into these systems also holds great promise for advancing precision medicine by improving model specificity and translational relevance. Notably, precise control of oxygen concentrations during hypoxia induction is critical, as varying oxygen levels can have diverse effects, depending on the context of the disease.

In this review, we underline the importance of integrating hypoxia and multilineage differentiation in the development of advanced organoid models that more accurately recapitulate organogenesis and pathological processes. Further research is essential to elucidate the role of hypoxia across various disease models, which will be instrumental in the design of more effective therapeutic strategies.

In conclusion, harnessing the synergistic interplay between hypoxia and multilineage communication offers significant promise for next-generation organoid platforms, positioning them as transformative tools in disease modeling and therapeutic discovery. Achieving this vision will require sustained interdisciplinary collaboration and the continuous refinement of organoid technologies.

## Abbreviations

The following abbreviations are used in this manuscript:

↑	upregulation
↓	downregulation
✓	Condition occurring
2D	Two-dimensional
3D	Three-dimensional
ALP	Alkaline phosphatase
Bio-P3	Bio-Pick, Place, and Perfuse
BM-MSCs	Bone marrow-derived mesenchymal stem cells
CL	Crypt-like
CNS	Central nervous system
CSC	Cancer stem cell
CVS	Cardiovascular system
ECM	Extracellular matrix
EMT	Epithelial–mesenchymal transition
bFGF	Basic fibroblast growth factor
HCC	Hepatocellular carcinoma
hESC	Human embryonic stem cells
HIF-α	Hypoxia-inducible factor-1-alpha
hiPSCs	Human induced pluripotent stem cells
HSC	Hematopoietic stem cells
hSIOs	Human small intestinal organoids
HSPCs	Hematopoietic stem/progenitor cells
iHeps	iPSC-derived human hepatocyte-like cells
IR	Ischemia–reperfusion
MSCs	Mesenchymal stem cells
OBs	Osteoblasts
PDAC	Pancreatic ductal adenocarcinoma
PDGF	Platelet-derived growth factor
PO_2_	Partial oxygen pressure
STAMP	Spheroid transfer assisted by magnetic printing
SWIFT	Sacrificial writing into functional tissues
TGF-β	Transforming growth factor-beta
TME	Tumor microenvironment
VEGF	Vascular endothelial growth factor
VL	Villus-like
vMPS	Vascularized microphysiological systems
VNQI	Vascular network quality index
